# Anatomic Subsites and Prognosis of Gastric Signet Ring Cell Carcinoma: A SEER Population-Based 1 : 1 Propensity-Matched Study

**DOI:** 10.1155/2022/1565207

**Published:** 2022-01-30

**Authors:** Yangyang Xie, Xue Song, Wenge Dong, Haimin Jin, Zhongkai Ni, Xiaowen Li, Hai Huang

**Affiliations:** ^1^Department of General Surgery, Hangzhou TCM Hospital Affiliated to Zhejiang Chinese Medical University, Hangzhou, Zhejiang Province 310000, China; ^2^Department of Pneumology, Hangzhou TCM Hospital Affiliated to Zhejiang Chinese Medical University, Hangzhou, Zhejiang Province 310000, China

## Abstract

**Background:**

The dismal prognosis of gastric signet ring cell carcinoma (GSRC) is a global problem. The current study is conducted to comprehensively evaluate clinicopathological features and survival outcomes in GSRC patients stratified by anatomic subsites. Then, predictive nomograms are constructed and validated to improve the effectiveness of personalized management.

**Method:**

The patients diagnosed with GSRC were recruited from the online SEER database. The influence of anatomic subsites on overall survival (OS) and cancer-specific survival (CSS) was evaluated using multivariate Cox regression and Kaplan-Meier analysis. Then, we employed propensity score matching (PSM) technique to decrease selection bias and balance patients' epidemiological factors. Predictive nomograms were constructed and validated. Sensitivity analysis was performed to validate the conclusion.

**Results:**

Multivariate Cox regression demonstrated that the patients with overlapping gastric cancer (OGC) suffered the highest mortality risk for OS (HR, 1.29; 95% CI, 1.23-1.36; *P* < 0.001) and CSS (HR, 1.33; 95% CI, 1.28-1.37; *P* < 0.001). Age, TNM stage, tumor localization, tumor size, surgery, and chemotherapy presented a highly significant relationship with OS and CSS. Following subgroup and PSM analysis, OGC patients were confirmed to have the worst OS and CSS. Then, nomograms predicting 6-month, 12-month, and 36-month survival were constructed. The area under the curve (AUC) value in ROC was 0.775 (95% CI, 0.761-0.793) for 6-month survival, 0.789 (95% CI, 0.776-0.801) for 12-month survival, and 0.780 (95% CI, 0.765-0.793) for 36-month survival in the OS group, while in the CSS group, it was 0.771 (95% CI, 0.758-0.790) for 6-month survival, 0.781 (95% CI, 0.770-0.799) for 12-month survival, and 0.773 (95% CI, 0.762-0.790) for 36-month survival.

**Conclusion:**

We identified anatomic subsites as a predictor of survival in those with GSRC. Patients with OGC suffered the highest mortality risk. The proposed nomograms allowed a relatively accurate survival prediction for GSRC patients.

## 1. Introduction

Gastric cancer (GC) is the fifth most frequently diagnosed malignancy and the third greatest cause of cancer-associated death worldwide [1]. Adenocarcinoma occupies the majority of GC [2]. Gastric signet ring cell carcinoma (GSRC) is a rare subtype of gastric adenocarcinoma, which is related to aggressive malignancy behavior and poor prognosis [3]. It is reported that the occurrence rate of GSRC has gradually risen in the past three decades in the United States [4].

Anatomically, the stomach is classified into two prime subsites: the proximal section, which is composed of the cardia and fundus, and the distal section, which includes the body, antrum, and pylorus. Some studies also include overlapping section, which denote that the tumor develops across two or more anatomic subsites [5]. Recently, it is demonstrated that cardia, noncardia, and overlapping GC have divergent biological features and predisposing factors, which should be considered separately to investigate GC behavior [6]. And subsite-specific analysis can promote targeting prevention and therapy. However, no risk stratification by anatomic subsites has been made in the patients diagnosed with GSRC before.

Herein, a population-based research was conducted to investigate the clinicopathological features and survival outcomes in GSRC incidence, stratified by anatomic subsites. And one-to-one propensity score matching (PSM) was made to examine the effect of tumor localization on GSRC prognosis. Besides, nomogram models to predict personal prognosis were constructed and validated based on multi-institution and multipopulation data from the Surveillance, Epidemiology, and End Result (SEER) database.

## 2. Materials and Methods

### 2.1. Patient Selection

**Figure 1:**
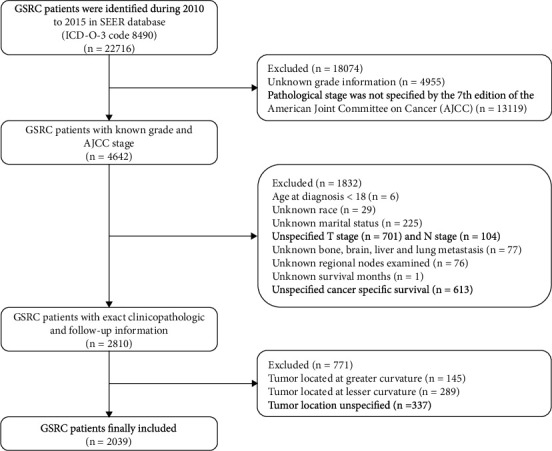


Patients were extracted from the SEER 18 regions' database (Incidence-SEER 18 Regs Research Data (with additional treatment fields), Nov 2017 Sub (1975-2016 varying)) using SEER∗Sat software (Version 8.3.5) [7]. We designed the following inclusion criteria: (1) age ≥ 18 years at diagnosis; (2) histology ICD-O-3 (International Classification of Diseases for Oncology, 3^rd^ edition) confined only to signet ring cell carcinoma (8490); and (3) patients with complete demographic, clinicopathological, treatment, and follow-up information. The detailed patient selection workflow is shown in Figure 1.

### 2.2. Clinicopathological Variables

Clinical features including tumor localization, age, race, marital status, gender, median household income, TNM grade, insurance status, tumor grade, T stage, N stage, M stage, tumor size, regional nodes examined, distal organic metastasis, treatment methods, and prognostic information were extracted for each patient. Based on the ICD-O-3 codes, anatomic subsites were characterized as follows: cardia (C16.0), fundus (C16.1), body (C16.2), antrum (C16.3), pylorus (C16.4), lesser curvature (C16.5), greater curvature (C16.6), overlapping (C16.8), and unspecified (C16.9), which was consistent with prior study [5]. Overlapping gastric cancer denoted that the tumor developed across two or more anatomic subsites. To avoid the inaccurate definition, only the vertical position classification (C16.0 to C16.4, C16.8) was included. As a result, the patients with tumor in cardia and fundus were divided into the proximal gastric cancer (PGC) group, while the tumors locating in corpus, antrum, and pylorus were included in the distal gastric cancer (DGC) group, and overlapping lesion of the stomach was in the overlapping gastric cancer (OGC) group. Age was categorized as 18-49 years, 50-59 years, 60-69 years, 70-79 years, and ≥80 years. Race was divided into black, white, American Indian/Alaska Native (AI), and Asian or Pacific islander (API). Median house income was categorized as quartile 1, quartile 2, quartile 3, and quartile 4 from bottom to top. TNM staging system was based on the 7^th^ edition of the American Joint Committee on Cancer (AJCC). Radiation therapy and chemotherapy were classified into “yes” and “no/unknown.” The study was exempted by institutional review boards due to the lack of subject identifiers and interventions.

### 2.3. Statistical Analysis

The categorical variables were tested using the chi-square test. The primary endpoints were overall survival (OS) and cancer-specific survival (CSS). Kaplan-Meier (KM) curves and log-rank test were used to estimate survival distribution. Cox proportional hazard models were applied to perform univariate and multivariate analyses. The proportional hazard assumption was assessed using Schoenfeld residuals and was met for all models (Figures S1 and S2).

PSM was a novel statistical method which could minimize the heterogeneity and mimic randomized controlled trials [8]. It was performed to reevaluate the impact of anatomic subsites using one-to-one nearest-neighbor matching and a caliper width of 0.01. Standardized difference (SD) was employed to examine the changes in covariate before and after PSM. SD ≤ 0.1 denoted significant balances in the baseline variables [9].

The predictive ability of nomograms was assessed by calibration curves and concordance index (*C*-index) [10, 11]. In the calibration plot, 1000 bootstrap resamples were conducted to investigate the consistence of the predicted and observed probabilities of survival. Besides, the receiver operating characteristic (ROC) curves were plotted to show the prediction power of the constructed model, and the area under the curve (AUC) value was listed. Higher AUC presented a stronger prediction power. Then, a sensitivity analysis was performed to validate the conclusion.

The statistical analyses were based on R software, version 4.0.3 (https://www.r-project.org) using packages of tableone, rms, survival, survminer, ggplot2, cobalt, pROC, and Matchit. A two-tailed *P* < 0.05 was indicated statistically significant.

## 3. Results

### 3.1. Clinicopathological Characteristics

A total of 2039 patients with GSRC from 2010 to 2015 were recruited in the SEER database. The clinicopathological features in each group are presented in Table 1.

The patients in the PGC group tended to be in the older age groups of 60-69 (31.1%) and 70-79 (20.5%). The PGC group had more white patients (82%), while there were more black (14.3%) and API (21.4%) patients in the DGC group. Male and female proportion was the highest in the PGC group (71.2%) and the DGC group (53.1%), respectively. Socioeconomic status was divided into quartile 1 (<$51030), quartile 2 ($51031-$61237), quartile 3 ($61238-$74330), and quartile 4 (>$74331). Compared to the OGC and PGC groups, the patients in the DGC group tended to have earlier stage (26.5%), T stage (27.4%), N stage (45.4%), M stage (76.2%) and smaller tumor size (17.3%). The OGC group presented the highest bone metastasis proportion (6.0%), but more liver metastasis (6%) and lung metastasis (4.5%) were found in the PGC group. The proportion of patients that underwent surgery presented the largest (67.1%) in the DGC group. And PGC patients had more intentions to receive radiation (49.3%) and chemotherapy (78.8%).

### 3.2. Effects of Tumor Localization on OS and CSS

**Figure 2:**
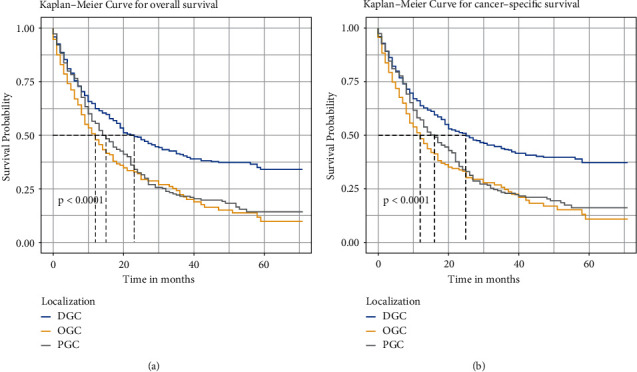


The OS and CSS of GSRC patients were evaluated by KM analysis. Significant differences in OS and CSS were found based on tumor localization (*P* < 0.0001) (Figure 2).

Univariate analysis demonstrated that tumor localization, age, race, marital status, median household income, TNM stage, tumor size, regional node examined, bone metastasis, liver metastasis, lung metastasis, surgery, radiation, and chemotherapy were significantly associated with OS (Table 2) and CSS (Table 3) (all *P* < 0.05).

The outcomes of multivariate Cox regression analysis demonstrated that the patients with DGC suffered relatively low risk for OS (OGC: HR, 1.29; 95% CI, 1.23-1.36; *P* < 0.001; PGC: HR, 1.15; 95% CI, 1.09-1.22; *P* < 0.001) (Table 2) and CSS (OGC: HR, 1.33; 95% CI, 1.28-1.37; *P* < 0.001; PGC: HR, 1.18; 95% CI, 1.10-1.23; *P* < 0.001) (Table 3). The result also presented that patients with OGC suffered the highest mortality risk. Age, TNM stage, tumor size, surgery, and chemotherapy presented high levels of correlation with OS and CSS.

**Figure 3:**
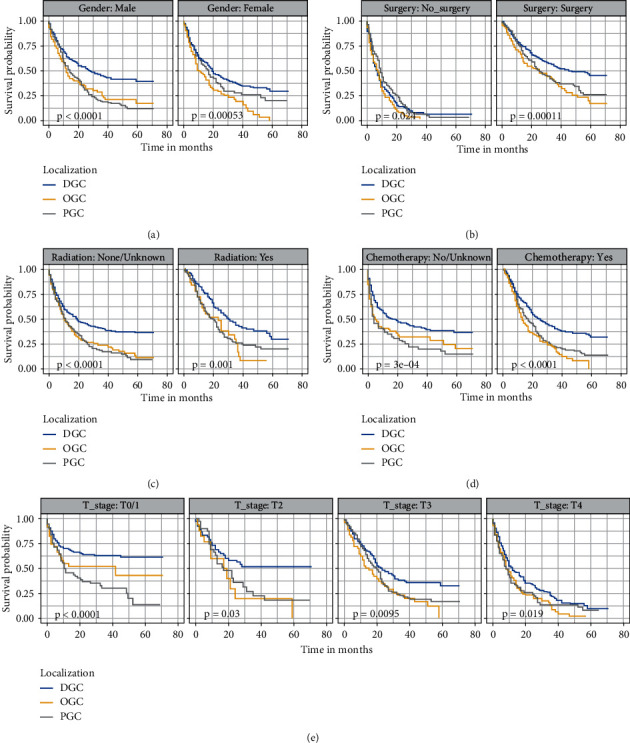


**Figure 4:**
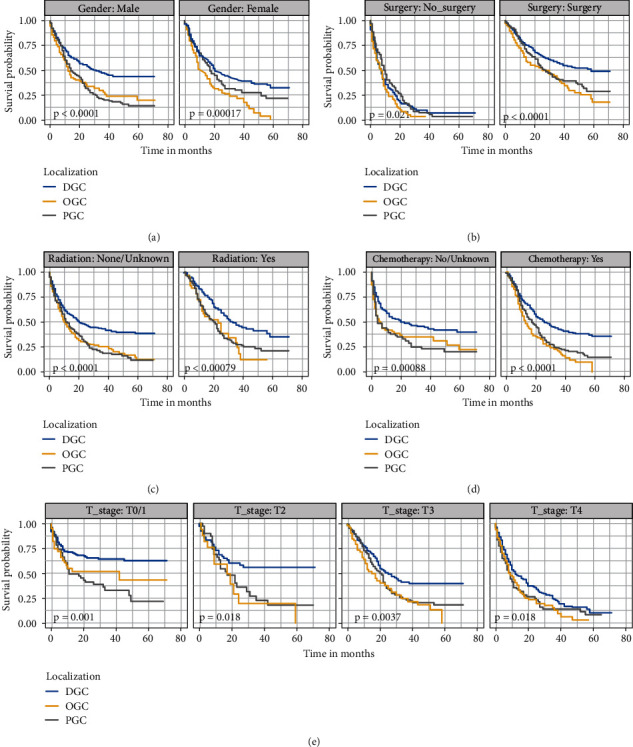


To decrease the impact of confounding factors, all GSRC patients were stratified based on clinical characteristics. It was identified that tumor localization was an independent prognostic factor of OS (Figure 3) and CSS (Figure 4) in the subgroups stratified by gender, surgery, radiation, chemotherapy, and T stage (all *P* < 0.05).

### 3.3. Survival Analysis after 1 : 1 PSM

**Figure 5:**
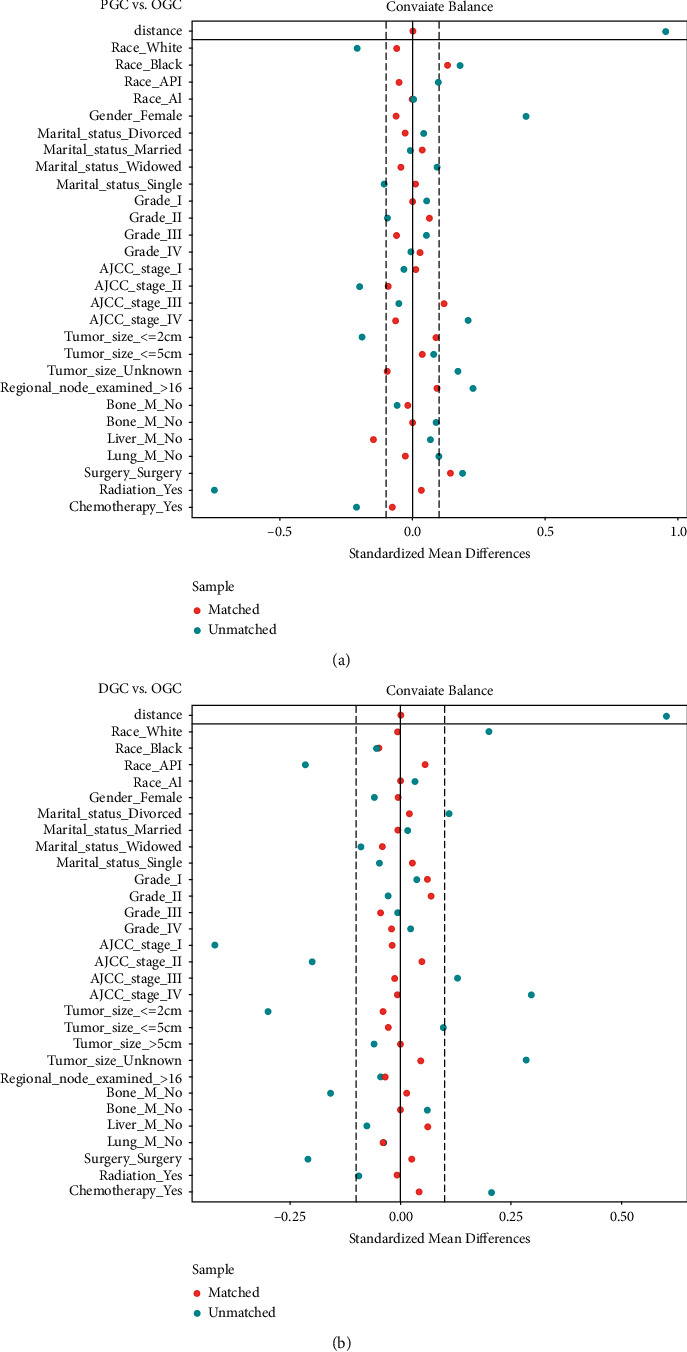


**Figure 6:**
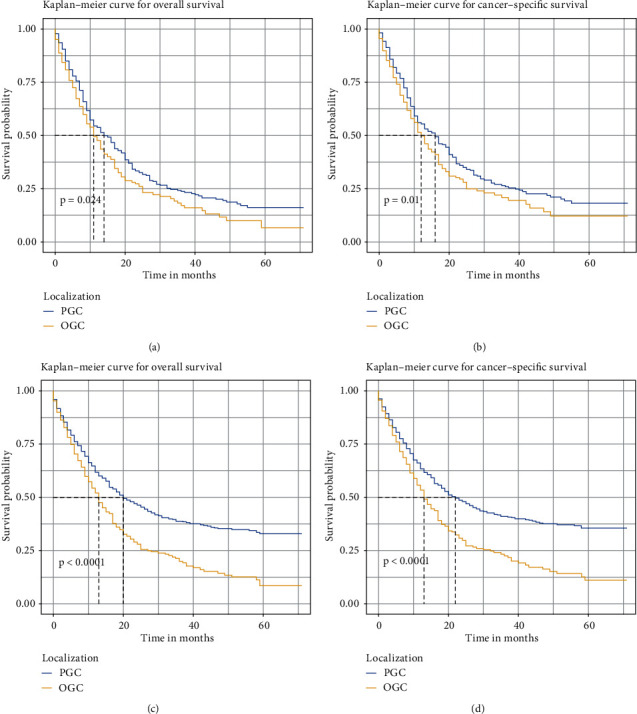


A one-to-one PSM was conducted to minimize the influence of potential confounders. Two matched groups were produced: a PGC and OGC cohort and a DGC and OGC cohort. The clinical baselines between both cohorts were balanced (Table 4). SD in most variables were less than 0.1, which indicated good balancing performance (Figure 5). OGC patients presented worse OS and CSS in the PGC-OGC cohort and the DGC-OGC cohort after PSM (Figure 6).

### 3.4. Construction and Validation of the Nomogram

**Figure 7:**
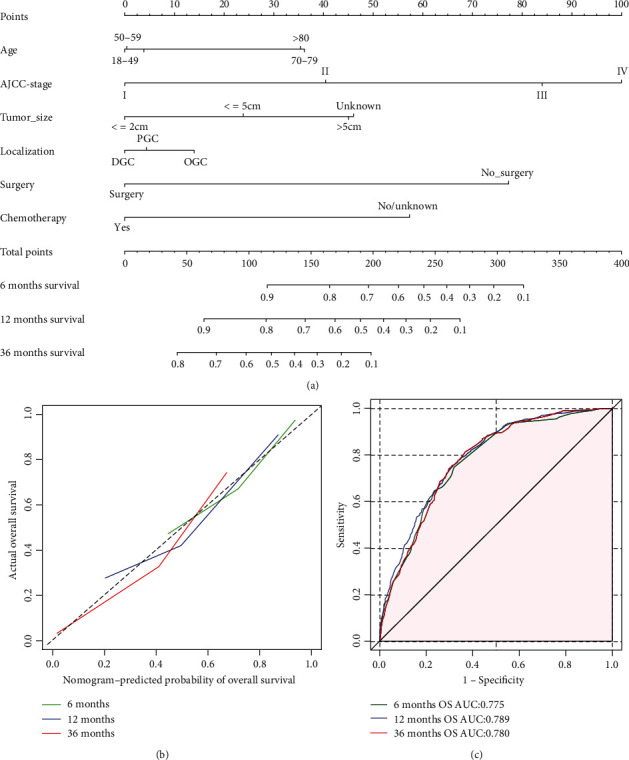


**Figure 8:**
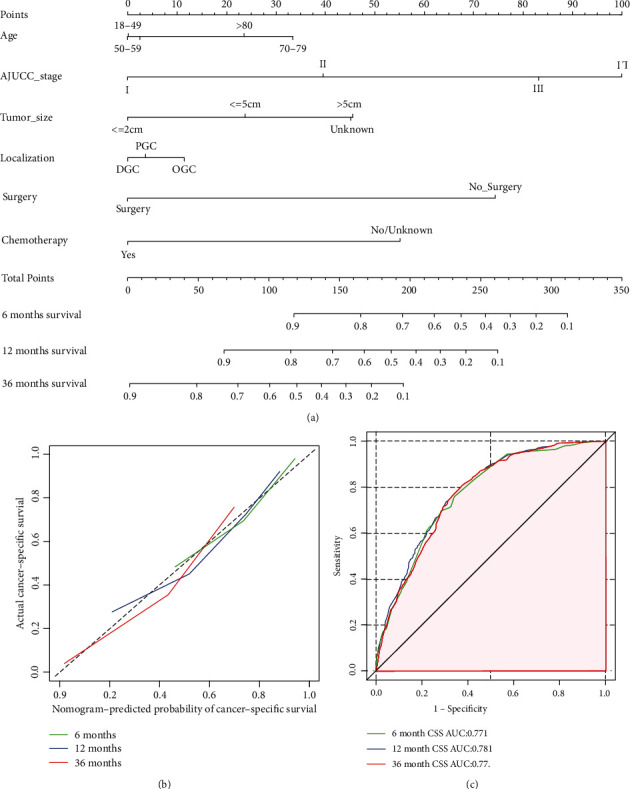


These six significant independent variables were applied to construct the prognostic nomograms to predict the 6-month, 12-month, and 36-month OS and CSS of GSRC patients: age, TNM stage, tumor size, tumor localization, surgery, and chemotherapy (Figures 7(a) and 8(a)).

The calibration curves for 6-month, 12-month, and 36-month OS and CSS showed good consistence between the predicted and observed probabilities of survival (Figures 7(b) and 8(b)). To measure the accuracy of the nomograms, the *C*-index of 0.751 (95% CI, 0.733-0.764) for OS and 0.764 (95% CI, 0.742-0.789) for CSS was concluded. Furthermore, the ROC curves regarding the predictive ability of 6-month, 12-month, and 36-month survival were constructed (Figures 7(c) and 8(c)). And the resulting AUC values were calculated. In the OS group, it was 0.775 (95% CI, 0.761-0.793) for 6-month survival, 0.789 (95% CI, 0.776-0.801) for 12-month survival, and 0.780 (95% CI, 0.765-0.793) for 36-month survival, respectively, while in the CSS group, it was 0.771 (95% CI, 0.758-0.790) for 6-month survival, 0.781 (95% CI, 0.770-0.799) for 12-month survival, and 0.773 (95% CI, 0.762-0.790) for 36-month survival, respectively.

### 3.5. Sensitivity Analysis

Considering that the tumor size was a high-risk factor and had a high proportion of unknown values, a sensitivity analysis was carried out to validate the conclusion. A total of 1354 patients were included with specified tumor size, and then, univariate and multivariate Cox proportional hazard was carried out, presenting that the patients with OGC suffered the highest risk for OS (HR, 1.27; 95% CI, 1.14-1.42; *P* < 0.001) (Table S1) and CSS (HR, 1.25; 95% CI, 1.11-1.38; *P* < 0.001) (Table S2). Moreover, in the KM analysis, patients with OGC suffered the worst survival in OS (*P* < 0.0001) and CSS (*P* < 0.0001) (Figure S3). Nomograms based on the six significant independent variables were constructed, and the relative calibration curves and ROC curves showed good consistence and predictive ability (Figure S4). These results confirm the conclusions above.

## 4. Discussion

GSRC is a highly malignant type of GC, with a reported 5-year survival rate of only 15.9% [12]. And it was identified that tumor location was correlated with GC behavior and patients' survival. Nevertheless, survival analysis of GSRC based on the tumor location continues to be scarce. Hence, it is urgent to make an in-depth study on the role of tumor location and establish a predictive model to guide better clinical practice. This was the first research to investigate the effect of tumor location on GSRC prognosis using PSM in the SEER database. The results confirmed the concept that PGC, DCG, and OGC were different malignant entities, which should be considered separately to improve GSRC incidence and verify driving risk factors.

Gender was an important factor influencing the occurrence of GC. In the research, the total ratio of males to females was 1.2 : 1, with a higher ratio (2.47 : 1) in the PGC patients, which might attribute to the unhealthy diet and habits in men, such as smoking or alcohol abusing [13]. In addition, this research showed that the PGC group presented to be more frequent in aging population, which was similar to previous Chinese reports [14, 15]. However, no correlation was found between age and tumor site in two European studies. The distinction might be partly due to the discrepancy of ethnic lines [16, 17]. In addition, there was a relatively higher frequency of AJCC stage IV (38.2%), N3 stage (30.5%), M1 stage (38.2%) patients in the OGC group, which suggested a more aggressive malignant behavior of OGC.

In multivariate Cox regression analysis, age, TNM stage, tumor size, tumor localization, surgery, and chemotherapy were identified as prognostic factors. The patients with OGC suffered the highest risk for OS (HR, 1.29; 95% CI, 1.23-1.36; *P* < 0.001) and CSS (HR, 1.33; 95% CI, 1.28-1.37; *P* < 0.001). Besides, it was well established that older age had lower survival time because of more comorbidities than the younger patients [18]. Furthermore, it was found that GSRC patients who received chemotherapy suffered lower risk, which was consistent with previous research [19]. Our results further supported former findings of larger tumor size as an independent prognostic role negatively correlated with GSRC patient survival. It was reported that larger tumor might present higher probability of invasive growth and lymph node metastasis [20, 21]. In most malignancy, histological grade was one of the indicators which determine prognosis. However, in this research, no significant correlation was found. Since approximately 97% of the GSRC patients were in the grade III/grade IV histological classification, grade failed to be a risk factor for determining patients' prognosis.

The prognosis in PGC and DGC still remained controversial. Majority of reports had demonstrated a significant poorer survival in PGC patients compared with DGC patients [14, 22–24], while no significant difference was found in other research [16, 25]. Katsuhiko et al. even reported a longer OS in patients with PGC [26]. The distinction might be associated with different staging and histology in different research [23, 24]. Furthermore, esophageal cancer was included into PGC in several studies, which led to confounding differences. However, few studies concerned the role of OGC. In our study, the subtypes of GC were confined to GSRC, and the anatomic subsites were clearly defined as DGC, OGC, and PGC. So the results were more convincing.

Before PSM, the results presented the best survival of DGC and the worst survival of OGC in OS and CSS. To minimize the influence of potential confounders, PSM was performed to balance the clinical baselines between both cohorts. We further confirmed that those who were diagnosed with OGC suffered worse OS and CSS in the PGC-OGC cohort and the DGC-OGC cohort. Thomassen et al. founded that between 1995 and 2011 in the Netherlands, primary cancer of overlapping location was associated with higher odds for gastric cancer peritoneal carcinomatosis and worse survival [27], which indicated that OGC presented more invasive features than DGC and PGC in part of GC patients. The overlapping tumor developed across two or more anatomic subsites, presenting more aggressive malignancy behavior than the one-site-confined tumor. The Cancer Genome Atlas (TCGA) Research Network recently identified four subtypes with different molecular profiles to classify GC: Epstein-Barr virus-positive, microsatellite unstable, genomically stable, and tumors with chromosomal instability [28]. The correlation between four molecular GC subtypes and different anatomical sites was observed, which presented that the majority of tumor arising at the proximal section were associated with chromosomal instability [29]. So the molecular profile of OGC should raise concern to explain the mechanism of invasiveness.

Nomograms had been regarded as efficient tools in clinical practice, which couldpredict numerical probabilities for individual patients by incorporating critical prognostic factors [30]. Many nomograms had demonstrated superiority over the traditional TNM staging system in predicting survival in multiple malignancies [31–34]. Several studies had constructed prognostic nomogram of patients with GSRC; however, these researches had either limited population selection or absence of accurate tumor location. Wei et al. only included locally advanced (stage II and stage III) GSRC and constructed CSS prognostic nomogram, finding that patients who received postoperative radiotherapy had a better prognosis than surgery alone [35]. Guo et al. enrolled GSRC patients from 2004 to 2015 and constructed nomogram, but tumor location was not included in the clinical variables [36]. Wang et al. enrolled nonelderly GSRC patients, and primary site was considered in the study. But the classification was ambiguous; even nonvertical position (lesser curvature) and body position were classified together. So the primary site did not present as an independent risk factor [37]. This was the first study to include all GSRC patients from 2010 to 2015 and clearly define anatomic subsites as DGC, OGC, and PGC. On the basis of the multivariate analysis results, age, TNM stage, tumor size, tumor localization, surgery, and chemotherapy were integrated into this predictive model. The calibration curves showed good consistence between the predicted and observed probability of survival. And the AUC values ranged from 0.773 to 0.789 in ROC curves, which showed high accuracy in predicting 6-month, 12-month, and 36-month OS and CSS. So the nomogram could present great prognostic efficiency among GSRC patients with different tumor locations.

Furthermore, a sensitivity analysis was performed to validate the results. A total of 1354 patients were included with specified tumor size. The results showed that the OGC patients still suffered the highest risk for OS (HR, 1.27; 95% CI, 1.14-1.42; *P* < 0.001) and CSS (HR, 1.25; 95% CI, 1.11-1.38; *P* < 0.001). And the constructed nomogram remained good consistence and predictive ability. The comprehensive statistical analyses adjusted for residual confounders, thus making the conclusion more reliable.

Limitation of the study includes the lack of detailed information of radiotherapy and chemotherapy, such as the dose of radiotherapy and the chemotherapy regimen. Also, this is a retrospective analysis, so selection bias is inevitably brought in. Further prospective research is necessary to confirm the conclusion. Despite these limitations, our substantial cases to investigate incidence by tumor location could provide novel insights on the epidemiology of GSRC.

## 5. Conclusion

In conclusion, we firstly identified anatomic subsites as a predictor of survival in those with GSRC. Patients with OGC suffered the highest mortality risk. The constructed nomograms presented a relatively good performance and could be considered a practical tool to predict personal prognosis in GSRC patients. Further studies should be conducted separately to learn more about the etiologies of GSRC based on the different anatomic subsites.

## Figures and Tables

**Table 1:** The characteristics of patients with GSRC according to tumor localization in the SEER database.

Characteristics		DGC	OGC	PGC	*P* value
		1084	351	604	
Age (%)	18-49	240 (22.1)	75 (21.4)	91 (15.1)	<0.001
	50-59	249 (23.0)	93 (26.5)	149 (24.7)	
	60-69	242 (22.3)	90 (25.6)	188 (31.1)	
	70-79	218 (20.1)	59 (16.8)	124 (20.5)	
	≥80	135 (12.5)	34 (9.7)	52 (8.6)	
Race (%)	White	691 (63.7)	255 (72.6)	495 (82.0)	<0.001
	Black	155 (14.3)	44 (12.5)	40 (6.6)	
	API	232 (21.4)	49 (14.0)	64 (10.6)	
	AI	6 (0.6)	3 (0.9)	5 (0.8)	
Gender (%)	Male	508 (46.9)	175 (49.9)	430 (71.2)	<0.001
	Female	576 (53.1)	176 (50.1)	174 (28.8)	
Marital status (%)	Divorced	99 (9.1)	45 (12.8)	69 (11.4)	0.009
	Married	646 (59.6)	212 (60.4)	367 (60.8)	
	Widowed	147 (13.6)	38 (10.8)	48 (7.9)	
	Single	192 (17.7)	56 (16.0)	120 (19.9)	
Median household income (%)	Quartile 1	268 (24.7)	94 (26.8)	183 (30.3)	0.017
	Quartile 2	299 (27.6)	83 (23.6)	128 (21.2)	
	Quartile 3	247 (22.8)	98 (27.9)	141 (23.3)	
	Quartile 4	269 (24.8)	76 (21.7)	152 (25.2)	
Insurance (%)	Insured	1025 (94.6)	335 (95.4)	587 (97.2)	0.126
	Uninsured	49 (4.5)	13 (3.7)	12 (2.0)	
	Unknown	10 (0.9)	3 (0.9)	5 (0.8)	
Grade (%)	I	1 (0.1)	1 (0.3)	0 (0.0)	0.663
	II	26 (2.4)	7 (2.0)	20 (3.3)	
	III	1033 (95.3)	334 (95.2)	568 (94.0)	
	IV	24 (2.2)	9 (2.6)	16 (2.6)	
TNM stage (%)	I	287 (26.5)	44 (12.5)	82 (13.6)	<0.001
	II	211 (19.5)	45 (12.8)	118 (19.5)	
	III	328 (30.3)	128 (36.5)	235 (38.9)	
	IV	258 (23.8)	134 (38.2)	169 (28.0)	
T stage (%)	T0/1	297 (27.4)	60 (17.1)	143 (23.7)	<0.001
	T2	134 (12.4)	28 (8.0)	66 (10.9)	
	T3	290 (26.8)	88 (25.1)	273 (45.2)	
	T4	363 (33.5)	175 (49.9)	122 (20.2)	
N stage (%)	N0	492 (45.4)	141 (40.2)	242 (40.1)	<0.001
	N1	237 (21.9)	59 (16.8)	223 (36.9)	
	N2	129 (11.9)	44 (12.5)	76 (12.6)	
	N3	226 (20.8)	107 (30.5)	63 (10.4)	
M stage (%)	M0	826 (76.2)	217 (61.8)	435 (72.0)	<0.001
	M1	258 (23.8)	134 (38.2)	169 (28.0)	
Tumor size (%)	≤2 cm	187 (17.3)	29 (8.3)	72 (11.9)	<0.001
	≤5 cm	320 (29.5)	53 (15.1)	185 (30.6)	
	>5 cm	257 (23.7)	116 (33.0)	135 (22.4)	
	Unknown	320 (29.5)	153 (43.6)	212 (35.1)	
Regional nodes examined (%)	≤16	706 (65.1)	236 (67.2)	471 (78.0)	<0.001
	>16	378 (34.9)	115 (32.8)	133 (22.0)	
Bone metastasis (%)	Yes	24 (2.2)	21 (6.0)	28 (4.6)	0.001
	No	1060 (97.8)	330 (94.0)	576 (95.4)	
Brain metastasis (%)	Yes	3 (0.3)	0 (0.0)	3 (0.5)	0.389
	No	1081 (99.7)	351 (100.0)	601 (99.5)	
Liver metastasis (%)	Yes	32 (3.0)	16 (4.6)	36 (6.0)	0.011
	No	1052 (97.0)	335 (95.4)	568 (94.0)	
Lung metastasis (%)	Yes	24 (2.2)	10 (2.8)	27 (4.5)	0.033
	No	1060 (97.8)	341 (97.2)	577 (95.5)	
Surgery (%)	No surgery	357 (32.9)	152 (43.3)	318 (52.6)	<0.001
	Surgery	727 (67.1)	199 (56.7)	286 (47.4)	
Radiation (%)	No/unknown	830 (76.6)	282 (80.3)	306 (50.7)	<0.001
	Yes	254 (23.4)	69 (19.7)	298 (49.3)	
Chemotherapy (%)	No/unknown	440 (40.6)	109 (31.1)	128 (21.2)	<0.001
	Yes	644 (59.4)	242 (68.9)	476 (78.8)	

**Table 2:** Impact of tumor localization on the OS by univariate and multivariate survival analysis before PSM.

Characteristics		Univariate analysis		Multivariate analysis		
		Log rank *χ*^2^	*P* value	HR	95% CI	*P* value
Tumor localization		49.4	<0.001			
	DGC			Reference		
	OGC			1.29	1.23-1.36	<0.001
	PGC			1.15	1.09-1.22	<0.001

Age		76.2	<0.001			
	18-49			Reference		
	50-59			1.01	0.84-1.22	0.886
	60-69			1.04	0.87-1.26	0.644
	70-79			1.72	1.41-2.11	<0.001
	≥80			1.71	1.35-2.17	<0.001

Race		12.3	0.007			
	White			Reference		
	Black			1.07	0.89-1.28	0.467
	API			0.89	0.75-1.06	0.192
	AI			1.12	0.57-2.20	0.733

Marital status		28.3	<0.001			
	Divorced			Reference		
	Married			0.98	0.81-1.19	0.858
	Widowed			1.04	0.81-1.34	0.758
	Single			1.08	0.86-1.35	0.508

Median household income		16.7	<0.001			
	Quartile 1			Reference		
	Quartile 2			0.91	0.81-1.02	0.102
	Quartile 3			1.06	0.94-1.19	0.319
	Quartile 4			0.89	0.79-1.00	0.047

TNM stage		437.0	<0.001			
	I			Reference		
	II			1.84	1.45-2.34	<0.001
	III			3.54	2.84-4.41	<0.001
	IV			4.22	3.34-5.32	<0.001

Tumor size		244.6	<0.001			
	≤2			Reference		
	≤5			1.47	1.14-1.88	0.002
	>5 cm			2.05	1.59-2.64	<0.001
	Unknown			2.01	1.57-2.58	<0.001

Regional node examined		122.1	<0.001			
	≤16			Reference		
	>16			0.83	0.70-0.99	0.041

Bone metastasis		81.6	<0.001			
	Yes			Reference		
	No			0.86	0.65-1.14	0.290

Liver metastasis		87.0	<0.001			
	Yes			Reference		
	No			0.88	0.68-1.13	0.309

Lung metastasis		74.3	<0.001			
	Yes			Reference		
	No			0.86	0.63-1.17	0.331
Surgery		527.7	<0.001			
	No			Reference		
	Yes			0.35	0.29-0.42	<0.001

Radiation		22.6	<0.001			
	No/unknown		Reference		
	Yes			0.98	0.84-1.14	0.801

Chemotherapy		11.0	<0.001			
	No/unknown		Reference		
	Yes			0.43	0.37-0.50	<0.001

OS: overall survival; PSM: propensity score matching; HR: hazard ratio; CI: confidence interval.

**Table 3:** Impact of tumor localization on the CSS by univariate and multivariate survival analysis before PSM.

Characteristics		Univariate analysis		Multivariate analysis		
		Log rank *χ*^2^	*P* value	HR	95% CI	*P* value
Tumor localization		47.0	<0.001			
	DGC			Reference		
	OGC			1.33	1.28-1.37	<0.001
	PGC			1.18	1.10-1.23	<0.001

Age		45.0	<0.001			
	18-49			Reference		
	50-59			1.00	0.83-1.21	0.972
	60-69			1.02	0.85-1.23	0.816
	70-79			1.70	1.39-2.09	<0.001
	≥80			1.46	1.14-1.88	0.003

Race		13.9	0.003			
	White			Reference		
	Black			1.01	0.84-1.22	0.896
	API			0.86	0.72-1.03	0.093
	AI			1.07	0.53-2.19	0.843

Marital status		16.8	<0.001			
	Divorced			Reference		
	Married			0.98	0.81-1.19	0.843
	Widowed			1.01	0.78-1.31	0.954
	Single			1.04	0.83-1.30	0.751

Median household income		15.7	0.001			
	Quartile 1			Reference		
	Quartile 2			0.90	0.80-1.02	0.090
	Quartile 3			1.05	0.93-1.19	0.388
	Quartile 4			0.90	0.79-1.01	0.080

TNM stage		471.1	<0.001			
	I			Reference		
	II			1.88	1.46-2.42	<0.001
	III			3.77	2.98-4.75	<0.001
	IV			4.62	3.62-5.90	<0.001

Tumor size		247.9	<0.001			
	≤2			Reference		
	≤5			1.50	1.15-1.96	0.003
	>5 cm			2.15	1.64-2.81	<0.001
	Unknown			2.07	1.59-2.69	<0.001

Regional node examined		116.8	<0.001			
	≤16			Reference		
	>16			0.85	0.71-1.02	0.074

Bone metastasis		83.4	<0.001			
	Yes			Reference		
	No			0.86	0.64-1.15	0.299

Liver metastasis		94.0	<0.001			
	Yes			Reference		
	No			0.88	0.68-1.14	0.321

Lung metastasis		74.2	<0.001			
	Yes			Reference		
	No			0.87	0.63-1.19	0.374
Surgery		525.4	<0.001			
	No			Reference		
	Yes			0.34	0.28-0.40	<0.001

Radiation		22.2	<0.001			
	No/unknown		Reference		
	Yes			0.97	0.83-1.13	0.707

Chemotherapy		6.5	0.010			
	No/unknown		Reference		
	Yes			0.42	0.36-0.49	<0.001

CSS: cancer-specific survival; PSM: propensity score matching; HR: hazard ratio; CI: confidence interval.

**Table 4:** Patients' baseline characteristics after PSM.

Characteristic		PGC	OGC	*P* value	DGC	OGC	*P* value
		220	220		310	310	
Age (%)	18-49	40 (18.2)	48 (21.8)	0.514	72 (23.2)	68 (21.9)	0.812
	50-59	59 (26.8)	60 (27.3)		74 (23.9)	81 (26.1)	
	60-69	57 (25.9)	60 (27.3)		70 (22.6)	73 (23.5)	
	70-79	49 (22.3)	35 (15.9)		66 (21.3)	56 (18.1)	
	≥80	15 (6.8)	17 (7.7)		28 (9.0)	32 (10.3)	
Race (%)	White	167 (75.9)	164 (74.5)	0.886	223 (71.9)	222 (71.6)	0.957
	Black	20 (9.1)	25 (11.4)		42 (13.5)	41 (13.2)	
	API	32 (14.5)	30 (13.6)		42 (13.5)	45 (14.5)	
	AI	1 (0.5)	1 (0.5)		3 (1.0)	2 (0.6)	
Gender (%)	Male	129 (58.6)	137 (62.3)	0.495	146 (47.1)	155 (50.0)	0.52
	Female	91 (41.4)	83 (37.7)		164 (52.9)	155 (50.0)	
Marital status (%)	Divorced	32 (14.5)	28 (12.7)	0.858	33 (10.6)	31 (10.0)	0.827
	Married	131 (59.5)	128 (58.2)		194 (62.6)	192 (61.9)	
	Widowed	19 (8.6)	20 (9.1)		30 (9.7)	37 (11.9)	
	Single	38 (17.3)	44 (20.0)		53 (17.1)	50 (16.1)	
Grade (%)	I	0	0	0.966	0 (0.0)	1 (0.3)	0.646
	II	3 (1.4)	3 (1.4)		5 (1.6)	7 (2.3)	
	III	210 (95.5)	209 (95.0)		299 (96.5)	294 (94.8)	
	IV	7 (3.2)	8 (3.6)		6 (1.9)	8 (2.6)	
TNM stage (%)	I	29 (13.2)	34 (15.5)	0.718	47 (15.2)	44 (14.2)	0.907
	II	35 (15.9)	29 (13.2)		39 (12.6)	44 (14.2)	
	III	76 (34.5)	71 (32.3)		115 (37.1)	110 (35.5)	
	IV	80 (36.4)	86 (39.1)		109 (35.2)	112 (36.1)	
Tumor size (%)	≤2 cm	31 (14.1)	23 (10.5)	0.439	26 (8.4)	29 (9.4)	0.937
	≤5 cm	35 (15.9)	41 (18.6)		51 (16.5)	53 (17.1)	
	>5 cm	70 (31.8)	62 (28.2)		103 (33.2)	105 (33.9)	
	Unknown	84 (38.2)	94 (42.7)		130 (41.9)	123 (39.7)	
Regional nodes examined (%)	≤16	160 (72.7)	162 (73.6)	0.914	196 (63.2)	206 (66.5)	0.449
	>16	60 (27.3)	58 (26.4)		114 (36.8)	104 (33.5)	
Bone metastasis (%)	Yes	13 (5.9)	16 (7.3)	0.701	7 (2.3)	7 (2.3)	1
	No	207 (94.1)	204 (92.7)		303 (97.7)	303 (97.7)	
Liver metastasis (%)	Yes	8 (3.6)	13 (5.9)	0.371	17 (5.5)	9 (2.9)	0.161
	No	212 (96.4)	207 (94.1)		293 (94.5)	301 (97.1)	
Lung metastasis (%)	Yes	8 (3.6)	9 (4.1)	1	10 (3.2)	9 (2.9)	1
	No	212 (96.4)	211 (95.9)		300 (96.8)	301 (97.1)	
Surgery (%)	No	101 (45.9)	105 (47.7)	0.774	123 (39.7)	129 (41.6)	0.683
	Yes	119 (54.1)	115 (52.3)		187 (60.3)	181 (58.4)	
Radiation (%)	No/unknown	160 (72.7)	165 (75.0)	0.664	245 (79.0)	251 (81.0)	0.616
	Yes	60 (27.3)	55 (25.0)		65 (21.0)	59 (19.0)	
Chemotherapy (%)	No/unknown	61 (27.7)	63 (28.6)	0.916	108 (34.8)	102 (32.9)	0.671
	Yes	159 (72.3)	157 (71.4)		202 (65.2)	208 (67.1)	

## Data Availability

The datasets generated and analyzed during the current study are available in the SEER database (https://seer.cancer.gov/) and from the corresponding authors upon reasonable request.

## Abbreviations

GC: Gastric cancer
GSRC: Gastric signet ring cell carcinoma
OS: Overall survival
CSS: Cancer-specific survival
PSM: Propensity score matching
SEER: Surveillance, Epidemiology, and End Result
PGC: Proximal gastric cancer
DGC: Distal gastric cancer
OGC: Overlapping gastric cancer
AI: American Indian/Alaska Native
API: Asian or Pacific islander
TNM: Tumor-node-metastasis
AJCC: American Joint Committee on Cancer
KM: Kaplan-Meier
HR: Hazard ratio
ROC: Receiver operating characteristic curves
AUC: Area under the curve.

## Supplementary Materials

Supplementary Materials Figure S1: Schoenfeld residuals' plot for overall survival. Figure S2: Schoenfeld residuals' plot for cancer-specific survival. Figure S3: overall survival (A) and cancer-specific survival (B) curves of selected patients based on tumor localization. Figure S4: validation of the nomogram composed of prognostic risk model. The calibration curve of the nomogram between the predicted probabilities of survival and the 45-degree line for overall survival (A) and cancer-specific survival (B). The area under the ROC curve was used to show the discrimination of the nomogram for overall survival (C) and cancer-specific survival (D). Table S1: impact of tumor localization on the OS by univariate and multivariate survival analysis in selected patients. Table S2: impact of tumor localization on the CSS by univariate and multivariate survival analysis in selected patients.
