# Supplementary material for: Anatomic Subsites and Prognosis of Gastric Signet Ring Cell Carcinoma: A SEER Population-Based 1 : 1 Propensity-Matched Study
Source: Biomed Res Int. 2022 Jan 30;2022:1565207. doi: 10.1155/2022/1565207 (PMC8818421; doi:10.1155/2022/1565207)
Supplement: Supplementary Materials — Figure S1: Schoenfeld residuals' plot for overall survival. Figure S2: Schoenfeld residuals' plot for cancer-specific survival. Figure S3: overall survival (A) and cancer-specific survival (B) curves of selected patients based on tumor localization. Figure S4: validation of the nomogram composed of prognostic risk model. The calibration curve of the nomogram between the predicted probabilities of survival and the 45-degree line for overall survival (A) and cancer-specific survival (B). The area under the ROC curve was used to show the discrimination of the nomogram for overall survival (C) and cancer-specific survival (D). Table S1: impact of tumor localization on the OS by univariate and multivariate survival analysis in selected patients. Table S2: impact of tumor localization on the CSS by univariate and multivariate survival analysis in selected patients. [file 1565207.f1.zip › Table S1.docx]

| Table S1. Impact of tumor localization on the OS by univariate and multivariate survival analysis in selected patients | | | | | | |
| --- | --- | --- | --- | --- | --- | --- |
| Characteristics | | Univariate analysis | | Multivariate analysis | | |
|  |  | Log rank χ² | P value | HR | 95% CI | P value |
| Tumor localization |  | 37.6 | <0.001 |  |  |  |
|  | DGC |  |  | Reference | |  |
|  | OGC |  |  | 1.27 | 1.14-1.42 | <0.001 |
|  | PGC |  |  | 1.12 | 1.07-1.31 | <0.001 |
| Age |  | 43.7 | <0.001 |  |  |  |
|  | 18-49 |  |  | Reference | |  |
|  | 50-59 |  |  | 0.93 | 0.73-1.19 | 0.577 |
|  | 60-69 |  |  | 1.11 | 0.88-1.42 | 0.402 |
|  | 70-79 |  |  | 1.72 | 1.33-2.23 | <0.001 |
|  | ≥80 |  |  | 1.68 | 1.22-2.32 | 0.001 |
| Race |  | 11.7 | 0.008 |  |  |  |
|  | White |  |  | Reference | |  |
|  | Black |  |  | 1.11 | 0.88-1.41 | 0.378 |
|  | API |  |  | 0.94 | 0.75-1.18 | 0.610 |
|  | AI |  |  | 0.87 | 0.34-2.19 | 0.764 |
| Marital status |  | 23.4 | <0.001 |  |  |  |
|  | Divorced |  |  | Reference | |  |
|  | Married |  |  | 0.82 | 0.63-1.05 | 0.115 |
|  | Widowed |  |  | 0.92 | 0.66-1.28 | 0.619 |
|  | Single |  |  | 0.99 | 0.74-1.34 | 0.973 |
| Median household income | | 18.6 | <0.001 |  |  |  |
|  | Quartile 1 |  |  | Reference | |  |
|  | Quartile 2 |  |  | 0.80 | 0.67-0.95 | 0.087 |
|  | Quartile 3 |  |  | 1.14 | 0.93-1.38 | 0.207 |
|  | Quartile 4 |  |  | 0.86 | 0.74-1.01 | 0.062 |
| TNM stage |  | 344.2 | <0.001 |  |  |  |
|  | I |  |  | Reference | |  |
|  | II |  |  | 2.79 | 1.94-4.01 | <0.001 |
|  | III |  |  | 6.11 | 4.34-8.61 | <0.001 |
|  | IV |  |  | 8.32 | 5.75-12.04 | <0.001 |
| Tumor size |  | 85.1 | <0.001 |  |  |  |
|  | ≤2 |  |  | Reference | |  |
|  | ≤5 |  |  | 1.18 | 0.91-1.53 | 0.211 |
|  | >5cm |  |  | 1.61 | 1.24-2.11 | <0.001 |
| Regional node examined | | 39.1 | <0.001 |  |  |  |
|  | ≤16 |  |  | Reference | |  |
|  | >16 |  |  | 0.80 | 0.66-0.96 | 0.091 |
| Bone metastasis |  | 64.0 | <0.001 |  |  |  |
|  | Yes |  |  | Reference | |  |
|  | No |  |  | 0.60 | 0.38-0.95 | 0.108 |
| Liver metastasis |  | 81.2 | <0.001 |  |  |  |
|  | Yes |  |  | Reference | |  |
|  | No |  |  | 0.87 | 0.61-1.26 | 0.464 |
| Lung metastasis |  | 43.1 | <0.001 |  |  |  |
|  | Yes |  |  | Reference | |  |
|  | No |  |  | 0.72 | 0.41-1.25 | 0.245 |
| Surgery |  | 264.0 | <0.001 |  |  |  |
|  | No |  |  | Reference | |  |
|  | Yes |  |  | 0.38 | 0.3-0.48 | <0.001 |
| Radiation |  | 3.94 | 0.04 |  |  |  |
|  | No/unknown | |  | Reference | |  |
|  | Yes |  |  | 0.93 | 0.77-1.12 | 0.444 |
| Chemotherapy |  | 2.1 | <0.001 |  |  |  |
|  | No/unknown | |  | Reference | |  |
|  | Yes |  |  | 0.51 | 0.42-0.62 | <0.001 |
| OS-overall survival; HR-hazard ratio; CI-confidence interval | | | | | | |
