# Supplementary material for: Anatomic Subsites and Prognosis of Gastric Signet Ring Cell Carcinoma: A SEER Population-Based 1 : 1 Propensity-Matched Study
Source: Biomed Res Int. 2022 Jan 30;2022:1565207. doi: 10.1155/2022/1565207 (PMC8818421; doi:10.1155/2022/1565207)
Supplement: Supplementary Materials — Figure S1: Schoenfeld residuals' plot for overall survival. Figure S2: Schoenfeld residuals' plot for cancer-specific survival. Figure S3: overall survival (A) and cancer-specific survival (B) curves of selected patients based on tumor localization. Figure S4: validation of the nomogram composed of prognostic risk model. The calibration curve of the nomogram between the predicted probabilities of survival and the 45-degree line for overall survival (A) and cancer-specific survival (B). The area under the ROC curve was used to show the discrimination of the nomogram for overall survival (C) and cancer-specific survival (D). Table S1: impact of tumor localization on the OS by univariate and multivariate survival analysis in selected patients. Table S2: impact of tumor localization on the CSS by univariate and multivariate survival analysis in selected patients. [file 1565207.f1.zip › Table S2.docx]

| Table S2. Impact of tumor localization on the CSS by univariate and multivariate survival analysis in selected patients | | | | | | | | | | | |  |
| --- | --- | --- | --- | --- | --- | --- | --- | --- | --- | --- | --- | --- |
| Characteristics | | Univariate analysis | | | | Multivariate analysis | | | | | |  |
|  |  | Log rank χ² | | P value | | HR | | 95% CI | | P value | |  |
| Tumor localization | | 40.1 | | <0.001 | |  | |  | |  | |  |
|  | DGC | |  | |  | | Reference | | | |  | |
|  | OGC | |  | |  | | 1.25 | | 1.11-1.38 | | <0.001 | |
|  | PGC | |  | |  | | 1.10 | | 1.05-1.27 | | <0.001 | |
| Age |  | | 27.1 | | <0.001 | |  | |  | |  | |
|  | 18-49 | |  | |  | | Reference | | | |  | |
|  | 50-59 | |  | |  | | 0.92 | | 0.72-1.18 | | 0.522 | |
|  | 60-69 | |  | |  | | 1.11 | | 0.86-1.41 | | 0.423 | |
|  | 70-79 | |  | |  | | 1.72 | | 1.32-2.25 | | <0.001 | |
|  | ≥80 | |  | |  | | 1.44 | | 1.02-2.02 | | 0.036 | |
| Race |  | | 12.8 | | 0.005 | |  | |  | |  | |
|  | White | |  | |  | | Reference | | | |  | |
|  | Black | |  | |  | | 1.08 | | 0.85-1.37 | | 0.541 | |
|  | API | |  | |  | | 0.91 | | 0.72-1.14 | | 0.407 | |
|  | AI | |  | |  | | 0.77 | | 0.28-2.16 | | 0.625 | |
| Marital status | | 14.6 | | 0.002 | |  | |  | |  | |  |
|  | Divorced | |  | |  | | Reference | | | |  | |
|  | Married | |  | |  | | 0.81 | | 0.62-1.05 | | 0.111 | |
|  | Widowed | |  | |  | | 0.88 | | 0.62-1.24 | | 0.456 | |
|  | Single | |  | |  | | 0.97 | | 0.72-1.32 | | 0.851 | |
| Median household income | | 18.6 | | <0.001 | |  | |  | |  | |  |
|  | Quartile 1 | |  | |  | | Reference | | | |  | |
|  | Quartile 2 | |  | |  | | 1.13 | | 0.92-1.39 | | 0.240 | |
|  | Quartile 3 | |  | |  | | 0.86 | | 0.73-1.01 | | 0.063 | |
|  | Quartile 4 | |  | |  | | 1.08 | | 0.87-1.35 | | 0.488 | |
| TNM stage | | 367.3 | | <0.001 | |  | |  | |  | |  |
|  | I | |  | |  | | Reference | | | |  | |
|  | II | |  | |  | | 3.11 | | 2.09-4.61 | | <0.001 | |
|  | III | |  | |  | | 7.15 | | 4.92-10.39 | | <0.001 | |
|  | IV | |  | |  | | 9.73 | | 6.73-15.13 | | <0.001 | |
| Tumor size | | 91.3 | | <0.001 | |  | |  | |  | |  |
|  | ≤2 | |  | |  | | Reference | | | |  | |
|  | ≤5 | |  | |  | | 1.18 | | 0.9-1.55 | | 0.242 | |
|  | >5cm | |  | |  | | 1.65 | | 1.25-2.18 | | <0.001 | |
| Regional node examined | | 36.2 | | <0.001 | |  | |  | |  | |  |
|  | ≤16 | |  | |  | | Reference | | | |  | |
|  | >16 | |  | |  | | 0.82 | | 0.67-1.00 | | 0.105 | |
| Bone metastasis |  | | 63.6 | | <0.001 | |  | |  | |  | |
|  | Yes | |  | |  | | Reference | | | |  | |
|  | No | |  | |  | | 0.63 | | 0.40-1.01 | | 0.073 | |
| Liver metastasis |  | | 84.7 | | <0.001 | |  | |  | |  | |
|  | Yes | |  | |  | | Reference | | | |  | |
|  | No | |  | |  | | 0.89 | | 0.62-1.29 | | 0.549 | |
| Lung metastasis |  | | 48.3 | | <0.001 | |  | |  | |  | |
|  | Yes | |  | |  | | Reference | | | |  | |
|  | No | |  | |  | | 0.69 | | 0.39-1.2 | | 0.184 | |
| Surgery |  | | 264.7 | | <0.001 | |  | |  | |  | |
|  | No | |  | |  | | Reference | | | |  | |
|  | Yes | |  | |  | | 0.37 | | 0.29-0.46 | | <0.001 | |
| Radiation |  | | 3.96 | | 0.042 | |  | |  | |  | |
|  | No/unknown | | | |  | | Reference | | | |  | |
|  | Yes | |  | |  | | 0.91 | | 0.74-1.10 | | 0.326 | |
| Chemotherapy | | 4.2 | | 0.021 | |  | |  | |  | |  |
|  | No/unknown | | | |  | | Reference | | | |  | |
|  | Yes | |  | |  | | 0.5 | | 0.41-0.62 | | <0.001 | |
| CSS-cancer-specific survival; PSM-propensity score matching; HR-hazard ratio; CI-confidence interval | | | | | | | | | | | |  |
