# Supplementary figures and images for: Anatomic Subsites and Prognosis of Gastric Signet Ring Cell Carcinoma: A SEER Population-Based 1 : 1 Propensity-Matched Study
Source: Biomed Res Int. 2022 Jan 30;2022:1565207. doi: 10.1155/2022/1565207 (PMC8818421; doi:10.1155/2022/1565207)

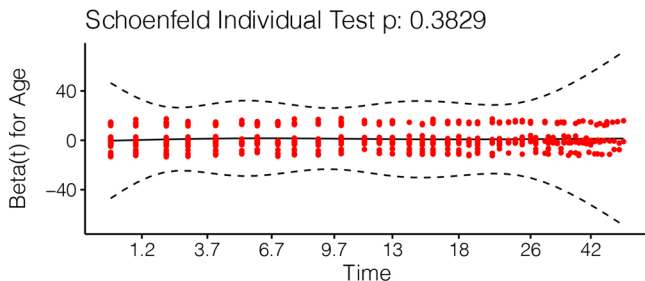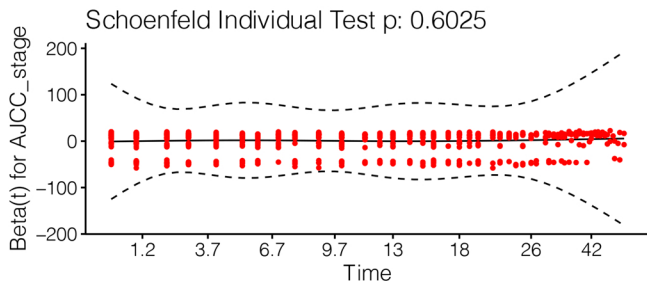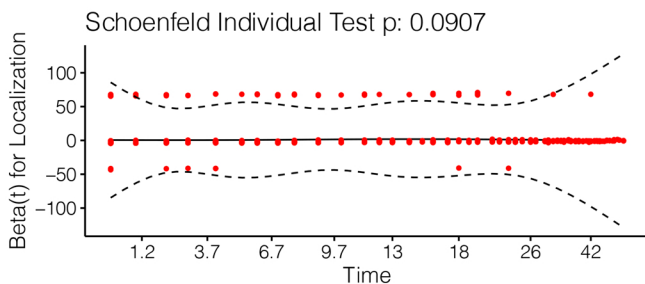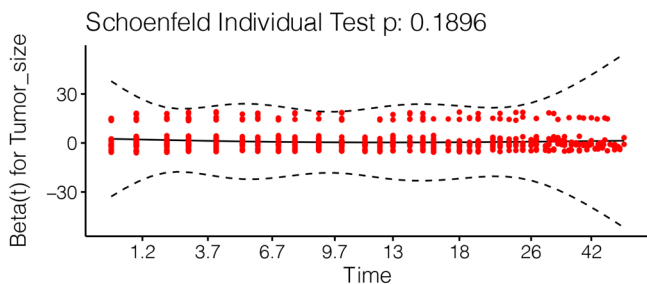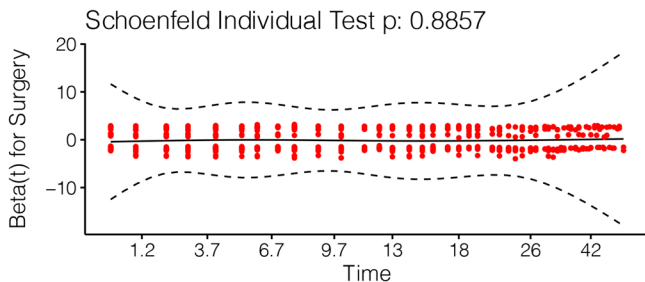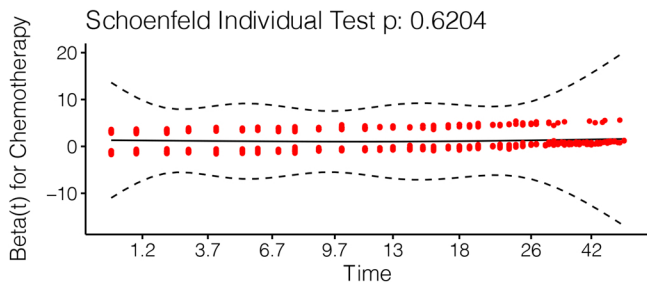

Supplement: Supplementary Materials — Figure S1: Schoenfeld residuals' plot for overall survival. Figure S2: Schoenfeld residuals' plot for cancer-specific survival. Figure S3: overall survival (A) and cancer-specific survival (B) curves of selected patients based on tumor localization. Figure S4: validation of the nomogram composed of prognostic risk model. The calibration curve of the nomogram between the predicted probabilities of survival and the 45-degree line for overall survival (A) and cancer-specific survival (B). The area under the ROC curve was used to show the discrimination of the nomogram for overall survival (C) and cancer-specific survival (D). Table S1: impact of tumor localization on the OS by univariate and multivariate survival analysis in selected patients. Table S2: impact of tumor localization on the CSS by univariate and multivariate survival analysis in selected patients. [file 1565207.f1.zip › Figure S1.pdf]

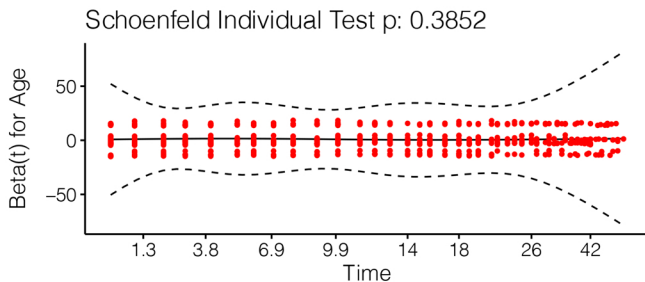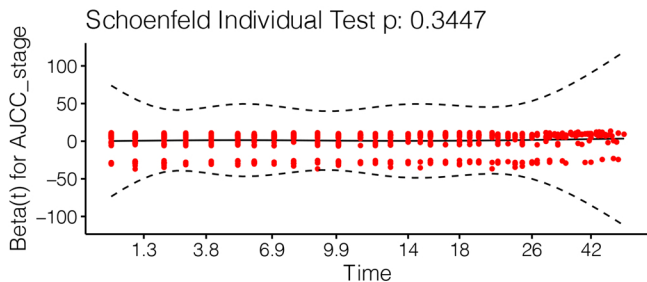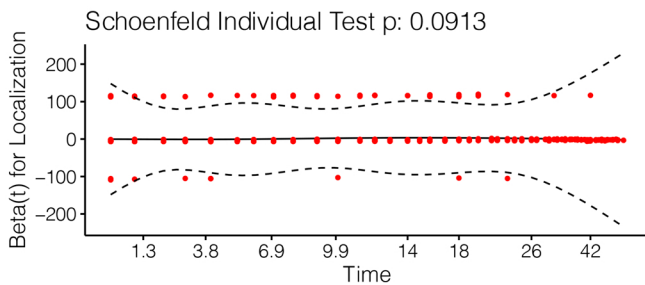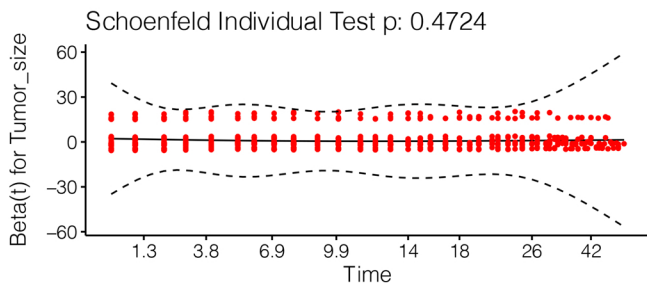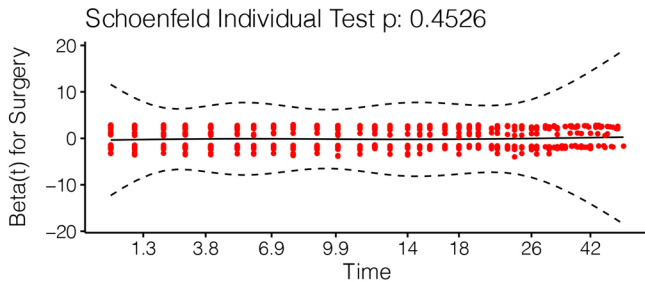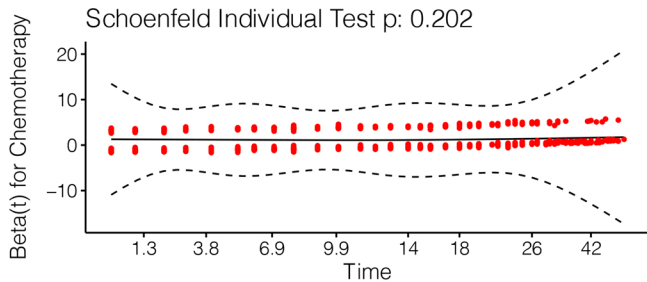

Supplement: Supplementary Materials — Figure S1: Schoenfeld residuals' plot for overall survival. Figure S2: Schoenfeld residuals' plot for cancer-specific survival. Figure S3: overall survival (A) and cancer-specific survival (B) curves of selected patients based on tumor localization. Figure S4: validation of the nomogram composed of prognostic risk model. The calibration curve of the nomogram between the predicted probabilities of survival and the 45-degree line for overall survival (A) and cancer-specific survival (B). The area under the ROC curve was used to show the discrimination of the nomogram for overall survival (C) and cancer-specific survival (D). Table S1: impact of tumor localization on the OS by univariate and multivariate survival analysis in selected patients. Table S2: impact of tumor localization on the CSS by univariate and multivariate survival analysis in selected patients. [file 1565207.f1.zip › Figure S2.pdf]

**A** Kaplan–Meier Curve for overall survival

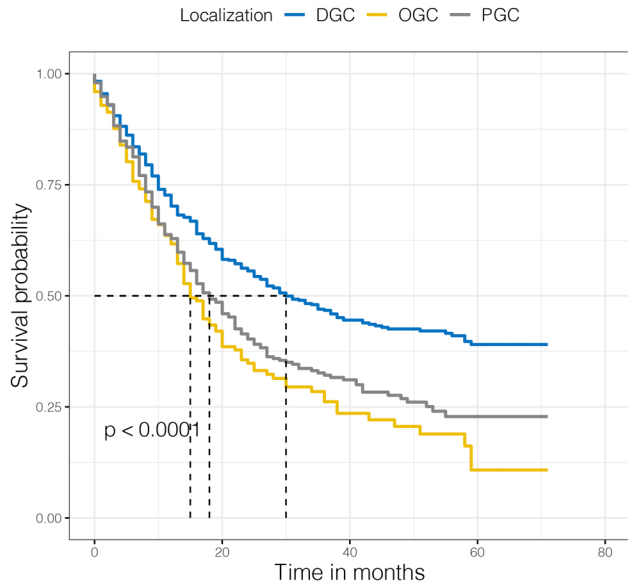

**B** Kaplan–Meier Curve for cancer-specific survival

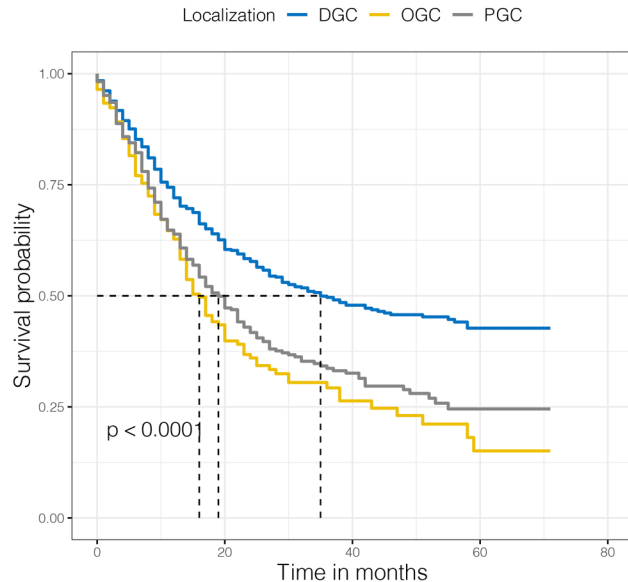

Supplement: Supplementary Materials — Figure S1: Schoenfeld residuals' plot for overall survival. Figure S2: Schoenfeld residuals' plot for cancer-specific survival. Figure S3: overall survival (A) and cancer-specific survival (B) curves of selected patients based on tumor localization. Figure S4: validation of the nomogram composed of prognostic risk model. The calibration curve of the nomogram between the predicted probabilities of survival and the 45-degree line for overall survival (A) and cancer-specific survival (B). The area under the ROC curve was used to show the discrimination of the nomogram for overall survival (C) and cancer-specific survival (D). Table S1: impact of tumor localization on the OS by univariate and multivariate survival analysis in selected patients. Table S2: impact of tumor localization on the CSS by univariate and multivariate survival analysis in selected patients. [file 1565207.f1.zip › Figure S3.pdf]

**A**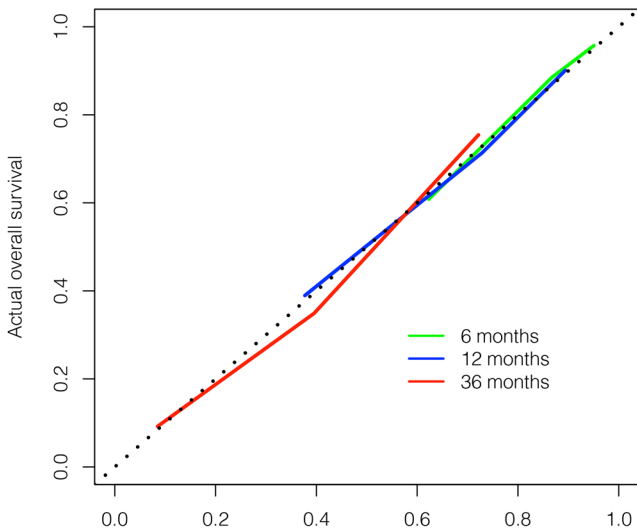**B**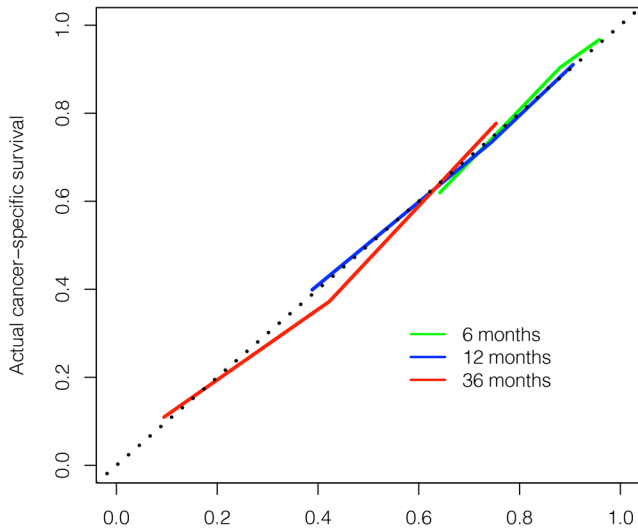**C**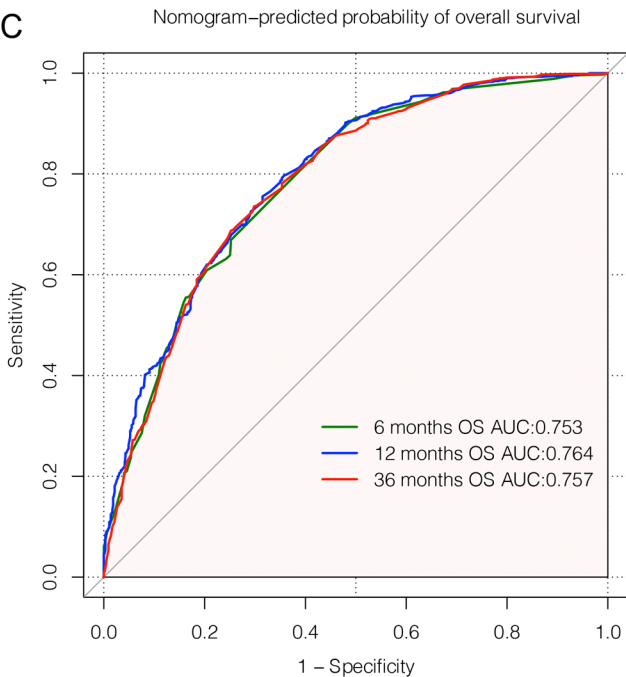**D**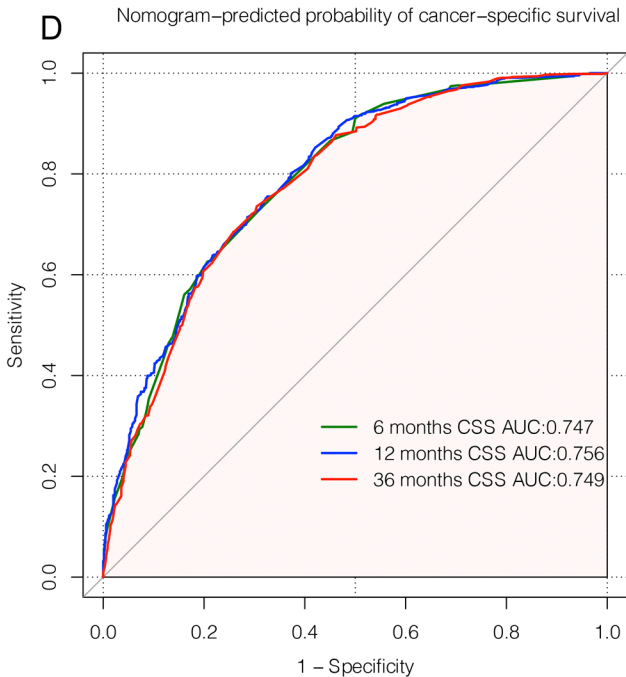

Supplement: Supplementary Materials — Figure S1: Schoenfeld residuals' plot for overall survival. Figure S2: Schoenfeld residuals' plot for cancer-specific survival. Figure S3: overall survival (A) and cancer-specific survival (B) curves of selected patients based on tumor localization. Figure S4: validation of the nomogram composed of prognostic risk model. The calibration curve of the nomogram between the predicted probabilities of survival and the 45-degree line for overall survival (A) and cancer-specific survival (B). The area under the ROC curve was used to show the discrimination of the nomogram for overall survival (C) and cancer-specific survival (D). Table S1: impact of tumor localization on the OS by univariate and multivariate survival analysis in selected patients. Table S2: impact of tumor localization on the CSS by univariate and multivariate survival analysis in selected patients. [file 1565207.f1.zip › Figure S4.pdf]
